# Ultrasensitive and Highly Selective Graphene-Based Field-Effect Transistor Biosensor for Anti-Diuretic Hormone Detection

**DOI:** 10.3390/s20092642

**Published:** 2020-05-06

**Authors:** Reena Sri Selvarajan, Ruslinda A. Rahim, Burhanuddin Yeop Majlis, Subash C. B. Gopinath, Azrul Azlan Hamzah

**Affiliations:** 1 Institute of Microengineering and Nanoelectronics (IMEN), National University of Malaysia (UKM), Bangi 43600, Malaysia; sreenat90@gmail.com (R.S.S.); burhan@ukm.edu.my (B.Y.M.); 2 Institute of Nanoelectronic Engineering (INEE), University Malaysia Perlis (UniMAP), Kangar 01000, Perlis, Malaysia; ruslinda@unimap.edu.my (R.A.R.); gopis11@gmail.com (S.C.B.G.)

**Keywords:** graphene FET, ADH-specific aptamer, anti-diuretic hormone, drain current, artificial kidney

## Abstract

Nephrogenic diabetes insipidus (NDI), which can be congenital or acquired, results from the failure of the kidney to respond to the anti-diuretic hormone (ADH). This will lead to excessive water loss from the body in the form of urine. The kidney, therefore, has a crucial role in maintaining water balance and it is vital to restore this function in an artificial kidney. Herein, an ultrasensitive and highly selective aptameric graphene-based field-effect transistor (GFET) sensor for ADH detection was developed by directly immobilizing ADH-specific aptamer on a surface-modified suspended graphene channel. This direct immobilization of aptamer on the graphene surface is an attempt to mimic the functionality of collecting tube $V_{2}$ receptors in the ADH biosensor. This aptamer was then used as a probe to capture ADH peptide at the sensing area which leads to changes in the concentration of charge carriers in the graphene channel. The biosensor shows a significant increment in the relative change of current ratio from 5.76 to 22.60 with the increase of ADH concentration ranging from 10 ag/mL to 1 pg/mL. The ADH biosensor thus exhibits a sensitivity of 50.00 µA·${\left(g/\mathrm{mL}\right)}^{-1}$ with a limit of detection as low as 3.55 ag/mL. In specificity analysis, the ADH biosensor demonstrated a higher current value which is 338.64 µA for ADH-spiked in phosphate-buffered saline (PBS) and 557.89 µA for ADH-spiked in human serum in comparison with other biomolecules tested. This experimental evidence shows that the ADH biosensor is ultrasensitive and highly selective towards ADH in PBS buffer and ADH-spiked in human serum.

## 1. Introduction

**Figure 1:**
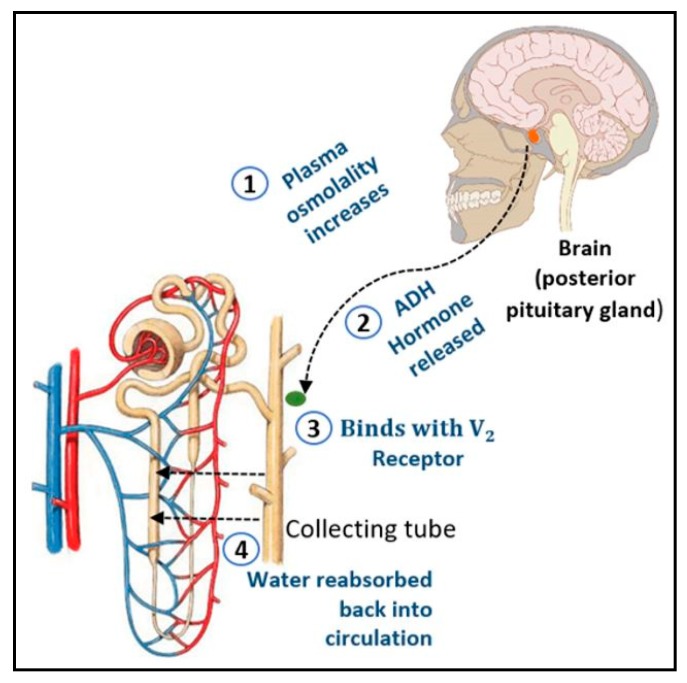
Role of anti-diuretic hormone (ADH) in maintaining plasma osmolality of human body.

Anti-diuretic hormone (ADH), also known as arginine-vasopressin or neurohypophysial hormone, is an imperative peptide for maintaining the plasma osmolality in the human body [1]. Theoretically, ADH is secreted by the hypothalamus and stored in the posterior pituitary gland, which will eventually release it into the bloodstream as plasma osmolality increases. The ADH will bind with $V_{2}$ receptors that are located at the basolateral membrane of collecting tube cells which will promote the reabsorption of water back into the circulation to maintain the water balance in the human body as illustrated in Figure 1 [2]. However, the failure of the kidney to respond to ADH will lead to excessive water loss from the body in the form of urine. This abnormal condition, which can be congenital or acquired, is termed nephrogenic diabetes insipidus (NDI) [3]. The kidney, therefore, has a crucial role in maintaining water balance and it is vital to restore this function in an artificial kidney. 

From 1950 to the present, several approaches have been developed and implemented for detecting ADH, namely bioassays, immunoassays, radioimmunoassays and enzyme-linked immunoassays (ELISAs). The extensive time consumption, extravagant procedure cost and low specificity of bioassays lead to the development of the immunoassay approach [4]. However, the implementation of immunoassay and radioimmunoassay demonstrated disadvantages due to the low molecular weight of the ADH hormone (1084.24 g/mol) and its poor immunogenic property [5]. The advent of the ELISA technique in ADH detection solves complications arising from previous techniques as it is more stable than a radiolabelled biomarker [6], does not use radioactive material [7] and uses less equipment to carry out the procedure [8]. The ELISA technique also has been implemented in other renal diseases such as quantification of plasma pentosidine [9] and detection of urinary podocalyxin in diabetic nephropathy patients [10]. 

Currently, there are many ADH assay kits available on the market, mostly dominated by ELISA-type kits such as the ADH/ vasopressin (VP)/ arginine-vasopressin (AVP) ELISA Kit from Biomatik. The lowest threshold exhibited by these kits is 0.5 pg/mL [11]. Although this threshold lies within the kidney’s ability to detect a normal ADH level, which is less than 1 $\mathrm{pg}\cdot \min^{-1}\cdot {\mathrm{kg}}^{-1}$ [12], the limitations of the ELISA technique mean it is not suitable to be applied for the detection of ADH in artificial kidney applications. This is because using the ELISA technique requires subtle sample handling and preparation procedures in laboratory conditions. This is time-consuming and it is not suitable for rapid diagnosis, particularly for ADH detection as ADH has a short half-life of 24 min [13]. Therefore, there is a high demand for an analytical tool that offers ultrasensitive detection of ADH with a lower limit of detection (LOD) of less than 1 pg/mL, highly specific and offers a rapid diagnosis with detection time less than the half-life of ADH peptide. 

One of the most promising tools to resolve these issues is the biosensor. Among the variety of electrical biosensing architectures reported [14,15], devices based on field-effect transistors have attracted much attention. A typical planar field-effect transistor (FET) consists of three metallic contacts which are the source (S), drain (D), and gate (G) electrodes, a thin insulating layer (dielectric) and the semiconductor substrate, the latter being the active part where charge carriers flow [16]. A comprehensive literature review reveals that graphene-based biosensors demonstrate ultrahigh sensitivity compared to one-dimensional1 carbon nanotubes (CNTs) [17]. The graphene’s high quality two-dimensional (2D) structure screens charge fluctuations, are highly sensitive to surrounding charges, offer higher charge carrier mobilities and have a very low limit of detection (LOD) compared to CNTs [18]. Therefore, incorporating a carbon nanomaterial, especially graphene, as the sensing material in the conducting channel hastens the opportunities for ultrasensitive, low-cost, low-noise and portable electrical biosensors for future device applications. 

A graphene-based field-effect transistor (GFET) with suspended graphene (the suspended graphene bridges the source and drain electrodes) holds more advantages than an embedded architecture (graphene supported by silicon dioxide substrate). This is because charge carriers in a suspended graphene FET offer high mobilities up to $10^{6\;}{\mathrm{cm}}^{2}V^{-1}s^{-1}$ in comparison with embedded GFET [19,20]. In addition, the absence of a supporting material in suspended GFET leads to the reduction of the signal-to-noise ratio (SNR) in the device and improves the transconductance (signal sensitivity) of the transducing element [21]. However, in embedded GFET, charges are trapped at the interface and the oxide layer acts as the external scattering centre [22]. This leads to a reduction in the transport properties of graphene. Hence, a suspended GFET with high carrier mobility is a suitable GFET architecture to be implemented for graphene-based biosensing applications. 

In this work, a FET-based biosensor was developed to detect ADH in experimental setting. A suspended graphene-based FET was fabricated for the ultrasensitive, selective and rapid detection of ADH. Moreover, ADH-specific aptamers were immobilized on the graphene channel as an attempt to mimic the functionality of the collecting tube $V_{2}$ receptors in the ADH biosensor. 

## 2. Materials and Methods

### 2.1. Field-Effect Transistor (FET) Fabrication and Graphene Transfer

Field-effect transistor (FET) devices were fabricated by using the conventional photolithography process and metal lift-off technique as described previously [23]. Single layer graphene (SLG) on copper foil purchased from Graphene Supermarket was used as the conducting channel material for the fabricated devices. Raman spectroscopy was carried out to authenticate the number of layers of the purchased graphene. The directional transfer technique was employed to transfer graphene onto pre-fabricated electrodes to bridge both the source and drain electrodes. This technique comprises of six major steps which are described as follows: (1) coating of poly (methyl methacrylate) (PMMA) on graphene layer; (2) etching of copper in iron (III) nitrate nanohydrate; (3) transferring PMMA-coated graphene onto substrate; (4) baking at 100 °C on a hotplate for 20 min; (5) removal of PMMA in an acetone bath for 30 min at 45 °C; (6) rinsing with isopropyl alcohol (IPA) and blow-drying the device. Finally, Raman spectroscopy was carried out to determine the quality of graphene by analysing changes in the structural and electronics properties of graphene. 

### 2.2. Functionalization of Graphene Surface in Graphene-Based Field-Effect Transistor (GFET)

Two microliters of 1 M of potassium hydroxide (KOH) with pH 9.0 acquired from Qrec (Asia) Sdn. Bhd. was pipetted onto the graphene layer at the channel area. A phosphate-buffered saline (PBS 10 mM, pH 7.4; Sigma Aldrich (St. Louis, MI, USA)) was used as the washing buffer. The device was characterized using attenuated total reflectance Fourier transform infrared (ATR-FTIR) spectroscopy to inspect for the successful chemical activation of the graphene layer. The process then proceeded with the functionalization of the graphene layer that comprises of two main stages which are surface modification and immobilization of aptamers.

The surface modification began with the introduction of 2% (3-aminopropyl) triethoxysilane (APTES) in 30% ethanol solvent to formulate an amine functionalized surface which was then left for 2 h at ambient condition. PBS buffer was used to rinse and wash the device three times to remove unbounded APTES residues from the surface. Subsequently, 2.5% glutaraldehyde (GA) was applied to the chemically modified graphene surface. This was left for an hour for the molecules to establish covalent bonding with the amine functionalized surface. The surface was then rinsed, washed and left till dry. After that, 1 µM of ADH specific-aptamer with the sequence of 5’ TCACGTGCATGATAGACGGCGAAGCCGTCGAGTTGCTGTGTGCCGATGCACGTGA-3’ [24] (purchased from Apical Scientific Sdn. Bhd.) was introduced at the channel area. 5’ end of this DNA aptamer is specially modified with an amine group to ensure the successful binding of aptamer on the graphene layer. The sample was left untouched for 15 min to allow the bonding of aptamers to the linkers, followed by the thorough washing of the surface with phosphate-buffered saline (PBS) three times. Then, 1 M of ethanolamine was deployed to block free space for 10 min and rinsed three times with the same buffer. The device was characterized using ATR-FTIR spectroscopy to inspect and validate the presence of functional groups after the functionalization with APTES, GA, and aptamer.

### 2.3. Anti-Diuretic Hormone (ADH) Detection

To depict the interaction between ADH peptide and aptamers, the GFET device was functionalized as mentioned in the previous section. ADH concentration ranging from 10 ag/mL to 1 pg/mL was titrated at the channel area of the functionalized GFET device. For each concentration, an incubation period of 10 min was allocated to allow the complete binding of aptamer and ADH protein. The surface was then washed with PBS buffer three times before proceeding with the next concentration of ADH. All the measurements were carried out by using Keithley 2400 Sourcemeter SMU Instruments.

### 2.4. Specificity Analysis

The surface-modified device was tested for the non-specific binding of blood-related biomolecules. Bovine serum albumin (BSA), urease, glucose oxidase and human serum without ADH hormone was independently applied at the channel area and electrical measurements were recorded using the Keithley 2400 Sourcemeter SMU Instruments. Similarly, human serum with ADH was tested to check for the specific functioning of the biosensor in human body conditions.

## 3. Results and Discussion

### 3.1. Surface and Morphological Characterization of GFET

**Figure 2:**
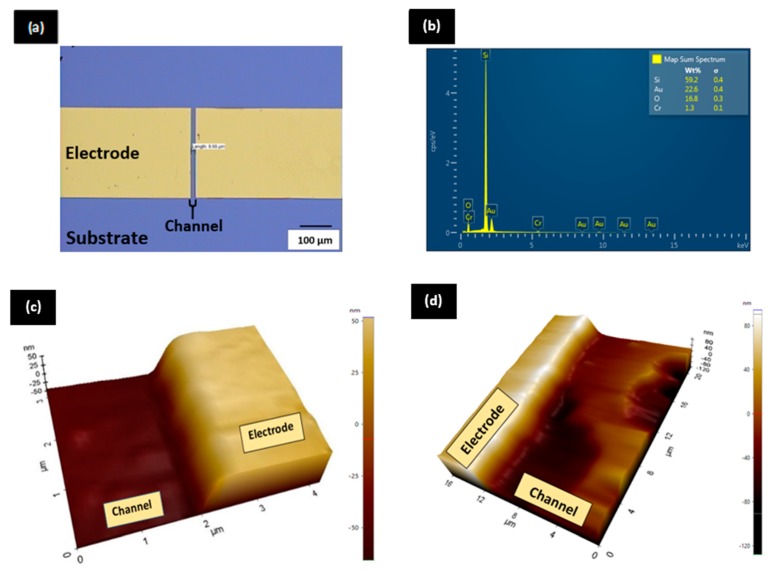
(**a**) Optical microscope image of graphene-based field-effect transistor (GFET) with 10 µm channel length; (**b**) energy-dispersive X-ray analysis (EDX) spectrum for GFET device; (**c**) atomic force microscopy (AFM) image for bare GFET and (**d**) AFM image of GFET after ADH detection.

The image of the fabricated device obtained from the optical microscope is illustrated in Figure 2a. This image depicts the 2D image of a bare GFET with 10 µm of channel length. However, the reading in Figure 2a shows 9.66 µm which is close to 10 µm. This shortage of 0.34 µm is due to error arising from the manual positioning of the ruler. Regardless of this, the image confirmed the fabrication of a complete unit of GFET with the desired channel length to be implemented in this work. 

The chemical composition of elements that made up the fabricated GFET was inspected and verified by performing the energy-dispersive X-ray analysis (EDX) on the GFET area comprising the source, drain and transducing channel. Figure 2b shows the percentage of elements present in the fabricated device with respective standard deviation presented clearly in the inset. The EDX spectrum exposes the presence of four distinct elements in the GFET which are silicon (Si), oxygen (O), chromium (Cr) and gold (Au). Silicon which is the substrate of GFET exhibits the highest proportion with 59.2%, followed by the electrode materials gold (22.6%) and chromium (1.3%), and finally oxygen (16.8 %) which represents the oxide layer. However, it is vital to take note that the presence of carbon that makes up the channel area was not detected by EDX spectroscopy. This is because the 15 kV E.H.T EDX spectroscopy was unable to quantify the presence of the very thin single layer of graphene, approximately 0.335 nm [25]. Instead, the presence of graphene at the channel area was verified with Raman spectroscopy which will be discussed in the subsequent section. The EDX spectrum in Figure 2b only verified that the GFET device is composed of Si, O, Cr and Au elements. 

The 3D image from the characterization of surface morphology by the atomic force microscope (AFM) is illustrated in Figure 2c. Based on the image obtained, the GFET device has a clear surface morphology. The uniform colour contrast at the channel area proved that the surface of graphene is bare and clean. The thickness between the bare channel area and the electrode is 60 nm as indicated by the scale. This also proved that the channel area is clean and bare. This verification of surface morphological characterization is essential to ensure that the surface is free from contamination before proceeding with the biosensing application. The characterization of the GFET surface after being utilized for ADH detection (Figure 2d) shows a drastic difference in the surface morphology in comparison with Figure 2c. The presence of a rough surface with an increase of thickness at the channel area validates the occurrence of ADH binding with ADH-specific aptamers. This change of surface morphology before and after ADH proves a successful detection of ADH at the channel area of GFET.

### 3.2. Raman Characterization for before and after Graphene Transfer

**Figure 3:**
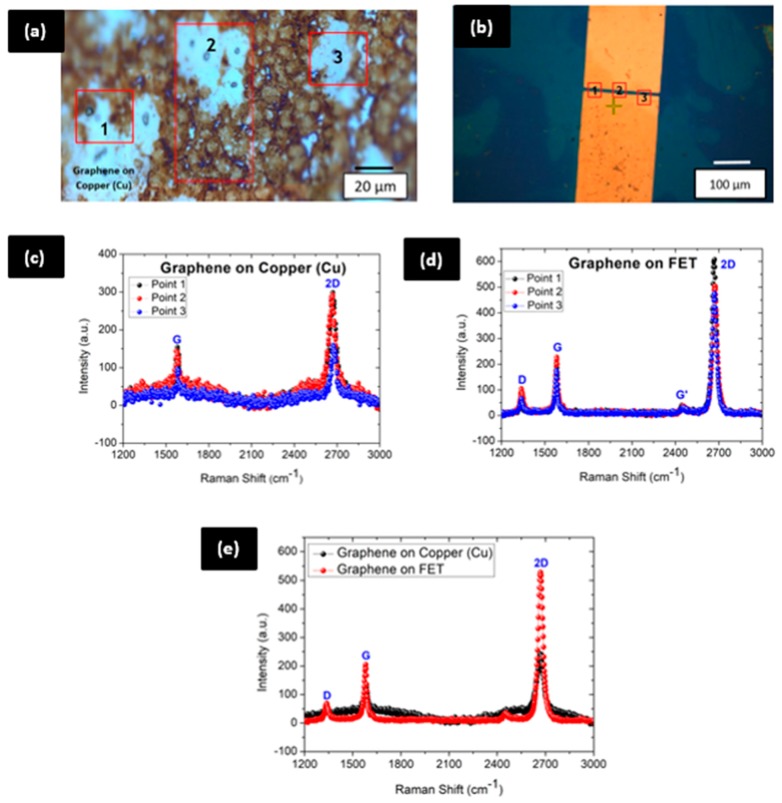
(**a**) Graphene on copper; (**b**) graphene on field-effect transistor (FET); (**c**) Raman spectrum for graphene on copper; (**d**) Raman spectrum for graphene on electrodes and (**e**) Raman spectrum (average value) for graphene on copper and FET.

Raman spectroscopy was performed to analyze the structural and electronic properties of the graphene before and after transferring onto the device (Figure 3). A 532 nm spot size of laser with 3.8 mW laser power was used to analyze the sample. Measurements were taken at three distinct points on the sample and graph of the average from all readings is shown in Figure 3e. The main features of Raman spectra which are G, D and 2D bands are located at 1350, 1580 and 2700 ${\mathrm{cm}}^{-1}$ respectively [26]. The ratio of the intensity of 2D to G ($I_{2D}/I_{G}$) used as a tool to indicate the number of layers in the graphene sample being tested. The data extracted from Raman spectroscopy for before and after the graphene transfer process are tabulated in Table 1 and Table 2, respectively.

#### 3.2.1. Raman Characterization for Graphene on Copper

Based on the data obtained and tabulated in Table 1, it is clear that the graphene on copper foil (Figure 3a) is made up of a combination of single and double layers. The ratios of $I_{2D}/I_{G}$ which are 1.92, 2.53 and 2.22 from the measurements indicate the presence of single and double-layer atomic structure of graphene in the sample purchased. It is also worth noting that the Raman spectrum shows the absence of a D-peak at 1350 ${\mathrm{cm}}^{-1}$ (Figure 3c). This provides a clear indication that the graphene is high quality, nearly perfect and defect-free. Moreover, graphene on copper foils is an excellent choice as the solubility of carbon atoms in copper is lesser compared to nickel [27]. Therefore, as indicated in Raman’s spectrum, it is viable to obtain a defect-free graphene sample on copper foil compared to graphene on nickel substrate. The frequency of the 2D peak also shows a significant shift towards the left which is reduced by 33.22 ${\mathrm{cm}}^{-1}$ (from 2700.00 ${\mathrm{cm}}^{-1}$ to 2666.78 ${\mathrm{cm}}^{-1}$). This shifting of the peak position in 2D band shows that the unit cell constant of graphene is enlarged or broadened as it has been grown on other substrates, such as copper, which creates unintentional doping [28,29]. 

#### 3.2.2. Raman Characterization for Graphene Transferred on Field-Effect Transistor (FET)

The image of the FET after graphene transfer is shown in Figure 3b. Raman measurements were carried out at three distinct points at the channel area. The Raman spectrum obtained from three readings and the average value obtained from all measurements were plotted in Figure 3d,e respectively. Graphene after the transferring process reveals a distinct D-peak in its Raman spectrum. This indicates the presence of a defect in the sample. A folding or crack in the graphene layer during the transfer process leads to the presence of a defect peak. However, the ratio of the intensity of D to G, $I_{D}/I_{G}$ from 0.19 till 0.46 which have been reported in Table 2 indicates that the graphene layer has undergone a minor defect and does not create major changes to the electronic properties of graphene. The values of $I_{2D}/I_{G}$ which are 2.84, 2.22 and 2.70 also indicate that the graphene is single layer. The narrow and more intense shape of the 2D band also supports the presence of single layer graphene [30]. However, there is a significant rise in the intensity of G and 2D bands after the transfer of graphene. This clearly shows that the steps involved in the transferring process introduce unintentional doping to the graphene layer. 

It is also worth mentioning that there is a sharp peak at 2450 ${\mathrm{cm}}^{-1}$, usually known as G* peak. The origin of this G* peak was firstly identified and reported by [31] in 2005. This similar trend of peak has been reported in works carried out by [32,33,34]. This peak originates from the double resonance Raman scattering process which was produced by the movement of two phonons around the k-point in the phonon dispersion process. The G* peak is known as a weak peak as the intensity of this peak is very small and can be eliminated by controlling the environmental factors such as turning off lights (conducting it in a dark or off-light environment) during measurements. Therefore, the Raman spectrum before and after graphene transfer revealed that the graphene used as the current conducting channel material is a single layer graphene with some minor defects introduced during the transfer technique (negligible as $I_{D}/I_{G}$ is very small).

### 3.3. Attenuated Total Reflectance Fourier Transform Infrared (ATR-FTIR) Characterization for Functionalization of Graphene

**Figure 4:**
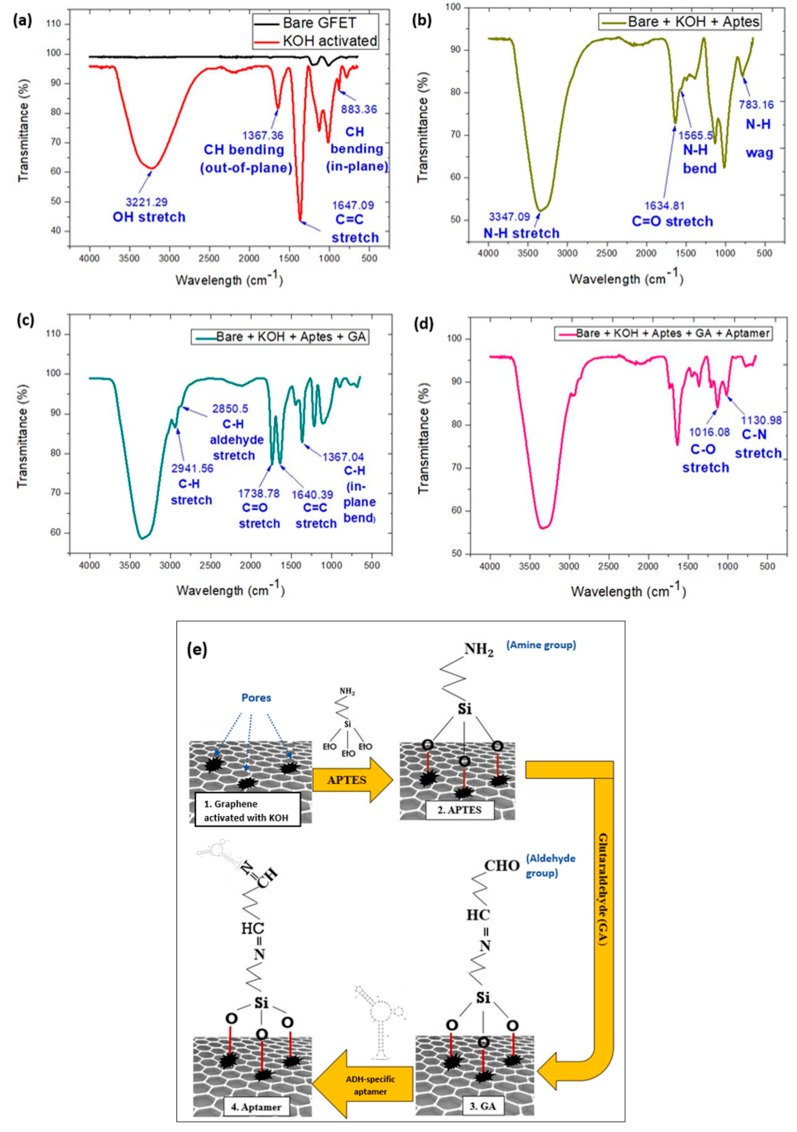
Attenuated total reflectance Fourier transform infrared (ATR-FTIR) spectra for surface functionalization of graphene in GFET. (**a**) KOH activation; (**b**) surface modification with APTES; (**c**) surface modification with glutaraldehyde (GA); (**d**) immobilization with ADH-specific aptamer and (**e**) schematic illustration of all stages in surface functionalization of graphene.

The ATR-FTIR transmission spectra of the graphene layer for four consecutive surface functionalization processes that begins with chemical activation (Figure 4a), surface modification with APTES (Figure 4b), GA (Figure 4c), and immobilization with ADH-specific aptamer (Figure 4d) are illustrated in Figure 4. Each stage introduces functional groups onto the graphene layer and the schematic illustration for the overall process is illustrated in Figure 4e.

Four new peaks emerged after the graphene layer was activated with 1 M of KOH in comparison with bare graphene (Figure 4a). A peak at 3221.29 ${\mathrm{cm}}^{-1}$ indicates a hydroxyl (OH) stretch occurred on the surface of graphene [35]. Valance vibration of hydrogens bonded to the hydroxyl (-OH) group indicated the presence and introduction of the oxygen-containing group onto the graphene layer. Besides that, a peak at 1647.06 ${\mathrm{cm}}^{-1}$ exhibits the C=C stretch. This shows the effect of KOH treatment on the double bond of the s$p^{2}$ lattice structure of graphene. Activation with KOH induces a defect in the structural arrangement of carbon lattice in graphene. A similar effect also was reported in work carried out by [36]. The peaks at 1367.36 ${\mathrm{cm}}^{-1}$ and 883.36 ${\mathrm{cm}}^{-1}$ represents in–plane CH bending [37] and out-of-plane CH bending vibrations, respectively [38]. Therefore, the ATR-FTIR characterization verified that activating graphene with KOH introduces oxygen-containing functional groups and causes a defect in its C=C lattice structure [39]. 

The KOH-activated graphene was then modified with APTES. From Figure 4b, it can be seen that three new peaks appeared, a medium peak at 783.16 c$m^{-1}$, a small peak at 1565.15 c$m^{-1}$ and a strong peak at 3347.09 c$m^{-1}$. Peaks at 783.16 c$m^{-1}$ and 1565.15 c$m^{-1}$ indicate the N-H wag and N-H bend respectively. The strong peak at 3347.09 c$m^{-1}$ represents amine N-H stretch which indicated the presence of amine in the functionalization step. The amine group binds with KOH-activated graphene via the silanization process and forms a strong bond of Si-O-metal. Therefore, these three peaks suggested the presence and successful binding of the free N$H_{2}$ group on the KOH-modified graphene surface. 

Subsequently, 2 µL of GA was added on the transducing layer. GA is a homo-bifunctional cross-linker with aldehyde groups on both ends with carbon chain spacer [40]. The aldehyde group from one side binds with the N$H_{2}$ group of APTES to form a secondary amide bond, while the aldehyde group from the other side is readily available for attachment with the N$H_{2}$ group from the ADH-specific aptamer bioreceptor. Thus, based on the results in Figure 4c, a strong peak of C=O stretch at 1738.78 c$m^{-1}$ confirms the emergence of the aldehyde group (-CHO) group from the functionalization step. The peaks in the range of 1600 c$m^{-1}$–1900 c$m^{-1}$, which are 1640.39 c$m^{-1}$ and 1738.78 c$m^{-1}$, demonstrate the establishment of an amide bond in between APTES and GA. This result of the ATR-FTIR shows strong agreement with a previous report [41]. Peaks associated with stretching at 2850.5 c$m^{-1}$ and 2941.56 c$m^{-1}$ which are C-H aldehyde and C-H stretches are fairly ubiquitous, and thus are omitted from determining the presence of a functional group. 

In the last stage, ADH-specific aptamer which is the bioreceptor for the ADH biosensor was immobilized on the surface-modified graphene. The measurement was taken only after 15 min to ensure the complete immobilization of ADH-specific aptamer on the surface-modified graphene at the channel area [42]. Based on the transmittance spectrum (Figure 4d), it can be seen that there are two distinct peaks related to stretching, which are 1016.08 and 1130.98, from this stage of functionalization. These peaks indicated the stretching of C-O and C-N bonds respectively. These bonds confirm the successful establishment of amide bonding between GA and ADH-specific aptamer. 

Thus, the successful surface modification and immobilization of ADH-specific aptamers on the KOH-modified graphene surface were verified through ATR-FTIR characterization. The schematic illustration of the stages involved in the surface functionalization of graphene is illustrated in Figure 4e.

### 3.4. Analytical Performance of GFET

**Figure 5:**
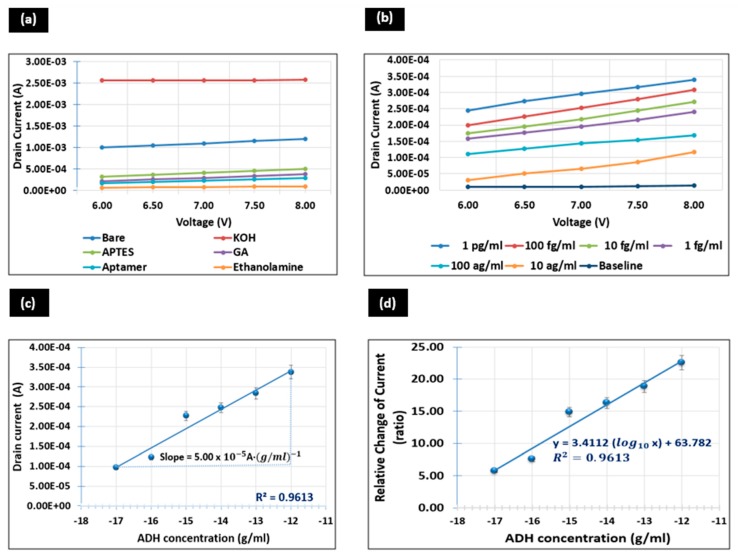
The current to voltage (I–V) measurements. (**a**) prior to surface functionalization; (**b**) for various ADH concentrations; (**c**) sensitivity and (**d**) limit-of-detection.

The electrical characteristics of the GFET device were monitored and measured as each individual surface functionalization step was carried out during the functionalization of the device (Figure 5a). Figure 5b, on the other hand, shows the current-voltage (I–V) curve for the interaction between immobilized ADH-specific aptamer and ADH spiked in PBS buffer. Following that, the sensitivity and LOD of the GFET device are illustrated in Figure 5c,d respectively. 

#### 3.4.1. I–V Curve for Surface Functionalization

3-point probe measurements were carried out and changes in drain current in the range of 6 to 8 V were observed (Figure 5a). At this range, graphene in the conducting channel behaves as an n-type channel material in which the majority charge carriers are electrons. At 8 V, bare graphene without any surface modification exhibits a drain current, $I_{D}$ of 1.20 mA. The addition of KOH onto the graphene channel leads to a drastic increase of $I_{D}$ to 2.58 mA. This is due to the presence and binding of ionic particles of KOH on graphene which leads to the increment of concentration of charge carriers in the graphene channel. Thus, graphene activated with KOH increases the conductivity of graphene. However, $I_{D}$ dropped from 2.58 mA to 508.85 µA after the addition of APTES on the transducing channel. The addition of a homobifunctional linker which is GA on the APTES-modified surface further reduces $I_{D}$ to 376.10 µA. Subsequently, the immobilization of ADH-specific aptamer on the surface-modified graphene channel causes a decrement in $I_{D}$ to 295.66 µA. 

The value of $I_{D}$ is only recorded in the 15th minute to ensure a complete immobilization of ADH-specific aptamer established on the modified surface. Finally, the addition of ethanolamine on the graphene channel leads to a reduction of $I_{D}$ to 94.81 µA. This value is only determined after 10 min of interaction in which $I_{D\;}$ exhibits a stable value that represents ethanolamine successfully blocking all free areas of the graphene layer. 

This drastic decrement of $I_{D}$ from bare graphene to aptamer-immobilized surface is due to the emergence of charge differences between each molecule when the surface functionalization process was carried out. Activation with KOH improves the conductivity of graphene but the functionalization with APTES, GA and immobilization with aptamer increases the resistance of the graphene channel. Therefore, the binding of each molecule on the graphene surface alters the surface chemistry of graphene which leads to a significant impact on the drain-to-source current of the GFET device. The results obtained here show good agreement with a previous work reported by [43]. 

#### 3.4.2. I–V Curve for Various ADH Concentrations

The drain current, $I_{D}$ for the interaction between immobilized ADH-specific aptamer and various ADH concentrations, between 10 ag/mL and 1 pg/mL, plotted as a function of gate voltage, $V_{G}$ with minimum drain bias, $V_{D}$ = 500 mV is shown in Figure 5b. The graph shows an increasing trend of $I_{D}$ as the concentration of ADH increases. ADH detection was begun by titrating the lowest ADH concentration which is 10 ag/mL and followed by 100 ag/mL. The titration of these two concentrations on the functionalized graphene surface exhibits $I_{D}$ of 116.26 µA and 167.91 µA at $V_{G}$ = 8 V respectively. Subsequently, the addition of 1 fg/mL, 10 fg/mL and 100 fg/mL of ADH concentration further increases the drain to 0.24045 mA, 0.27154 mA and 0.30842 mA, respectively. Each time before proceeding with the subsequent concentration, the transducing channel area was rinsed and washed with PBS buffer to ensure the binding is selective to the particular concentration of the target. Finally, titration with the highest concentration, which is 1 pg/mL gives rise to the highest drain of 0.33864 mA. 

The increase of drain current in conjunction with the increase of ADH concentration clearly authenticates that the binding of positively charged ADH hormone with ADH-specific aptamer increases the number of positively charged biomarkers at the surface of the transducing channel. This, in turn, exerts an attractive force to attract negatively-charged charge carriers to accumulate in the channel area. Thus, the concentration of electrons increases in the transducing channel area (the area between the source and drain electrodes). This, in turn, increases the conductivity of graphene which is proved by the increase of drain current in the biosensor. This increase in ADH concentration leads to the increase of concentration of charge carriers in the transducing channel which causes a significant increase in $I_{D}$ of GFET. 

#### 3.4.3. Sensitivity

The graph of Figure 5c portrays the drain current at 8 V versus the logarithmic concentration of ADH spiked in PBS. The readings were taken three times to obtain an average value (n = 3) for each concentration and error bars were plotted from the standard deviation of the values obtained. From visual inspection and stronger correlation coefficient data, the attainment of a linear relationship between $I_{D}$ and the increasing logarithmic ADH concentrations ranging from 10 ag/mL to 1 pg/mL is pretty clear. The sensitivity of the ADH biosensor was determined from the slope of the calibration curve plotted (Figure 5c). The linear regression of this curve is $I=5.00e^{-5}$ ($\log_{10}$ *c* + 0.0009) with a correlation coefficient of 0.9613. Therefore, based on the slope of the calibration curve, the sensitivity of this biosensor to detect ADH spiked in PBS buffer is 5.00 × $10^{-5}$ A·${\left(g/\mathrm{mL}\right)}^{-1}$. 

#### 3.4.4. Limit of Detection (LOD)

The graph of the ratio of relative change in drain current versus logarithmic concentration of ADH spiked in PBS buffer is illustrated in Figure 5d. The linear fit assumes a regression coefficient of $R^{2}$ = 0.9613 in the concentration ranging from 10 ag/mL to 1 pg/mL. This shows that 96.13 % of the relative change in $I_{D}$ is influenced by the logarithmic concentration of ADH. 

The limit of detection (LOD) was considered with the lowest concentration of an analyte (from the calibration line at low concentrations) against the background signal (S/N = 3:1), in other words, LOD = standard deviation of the baseline + 3σ. Based on 3σ calculation, the ADH biosensor exhibits a limit of detection as low as 3.55 ag/mL. LOD in the range of $10^{-18}$ (attomolar) illustrates the effectiveness of this biosensor for detecting ADH peptides in lower concentrations. This is due to the architecture of suspended graphene in GFET that exhibits high mobility and is highly sensitive to the surface charges which demonstrate the effectiveness of ADH detection in very low concentrations. The KOH-modified graphene also improves the sensitivity of the graphene layer by increasing its specific surface area. 

Therefore, LOD as low as 3.55 ag/mL shows an improvement in the detection limit compared to the research work that has been previously reported [44,45,46]. The biosensor’s ability to detect ADH below 1 pg/mL restores the functionality of the kidney in the GFET sensor to detect ADH. Therefore, this work demonstrated laboratory evidence of the GFET biosensor for ultrasensitive detection of ADH which encourages further in vivo testing within the human body. 

### 3.5. Specificity Analysis

#### 3.5.1. Specificity Analysis for ADH Spiked in Phosphate-Buffered Saline (PBS) Buffer

**Figure 6:**
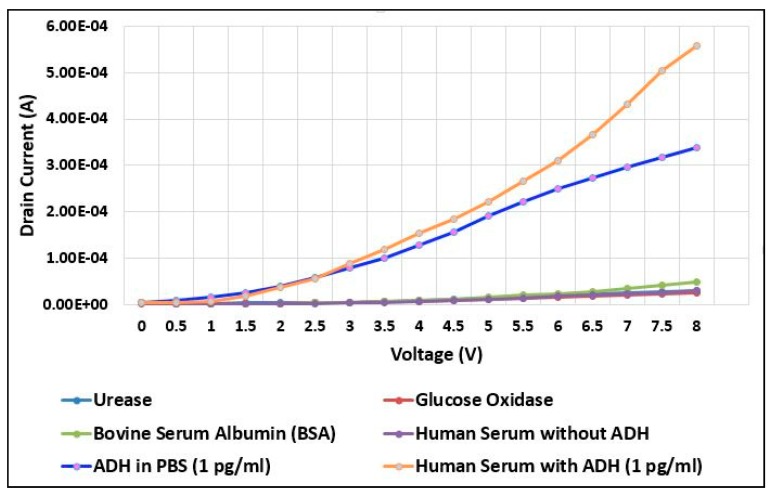
Specificity analysis of ADH biosensor tested with ADH spiked in phosphate-buffered saline (PBS) buffer, ADH spiked in human serum and other biomolecules.

The specificity analysis on the ADH biosensor was carried out by dropping different biomolecules independently on the ADH-specific aptamer-immobilized transducing channel (Figure 6). The graph shows the interaction of 1 pg/mL of ADH spiked in PBS buffer with ADH-specific aptamers reported at $I_{D}$ of 338.64 µA. This drain current value was kept as a benchmark to understand the interaction of ADH-specific aptamer with other biomolecules. 

Glucose oxidase (GOx) is an enzyme conventionally found in the human body, whereas urease is also an enzyme that could not be synthesised by mammalian cells. However, it can be produced by myriad bacteria found in the body, particularly in the intestine [47]. The interaction of GOx and urease on the ADH-specific aptamer exhibits a drain current of 25.55 µA and 30.73 µA respectively. The recorded little amount of drain indicated that the interaction was non-specific and strongly proved that ADH biosensor is highly selective in binding with its specific target. Subsequently, 1 pg/mL BSA was dropped on the transducing channel and the changes in the drain current were recorded. It can be observed that drain current increased to 47.34 µA which was slightly higher compared to the drain current obtained from the interaction of GOx and urease. However, this also indicated that the drain current has a drastic gap difference with the drain current obtained from 1 pg/mL of ADH spiked in PBS buffer. Therefore, this clearly indicates that the ADH biosensor is highly selective and specific to ADH. 

#### 3.5.2. Specificity Analysis for ADH-Spiked in Human Serum

Finally, human serum without ADH was pipetted on the functionalized transducing channel and the drain current was recorded. Based on Figure 6, it can be seen that the drain current was documented to be only 29.53 µA. This value has a drastic gap of drain current in comparison with the drain current obtained from ADH spiked in PBS buffer. This proved that the absence of ADH in the human plasma and the interaction was non-specific as there was no significant change in drain current and carrier charge density at the transducing channel area. This is also evident by the fact that ADH is only released by the hypothalamus and streamed into the blood as there is a need to conserve water level in the human body. Therefore, blank human serum does not bring any significant change in the conductance of the biosensor as the interaction is not specific. 

When 1 pg/mL of ADH spiked with human serum was tested on the ADH-specific aptamer immobilized transducing channel, the drain current reading was recorded after a wait of 10 min. The drain current was then increased up to 557.89 µA when it indicated the successful binding of ADH-specific aptamer with ADH-spiked in human serum. This strongly suggested and proved that ADH biosensor is highly specific and selective. ADH spiked in PBS buffer and human serum demonstrated a large change in current and recorded a higher current compared to other tested biomolecules. This shows that specific interaction only takes place with ADH-spiked in PBS buffer and ADH-spiked in human serum which increases the charge carrier density at the transducing channel and eventually leads to a drastic change in current. Therefore, the ADH biosensor is highly selective and specific in interacting with ADH molecules which is promising for future applications in an artificial kidney.

## 4. Conclusions

In conclusion, the ADH biosensor has a sensitivity of 50.00 µA·${\left(g/\mathrm{mL}\right)}^{-1}$ and a very low detection limit (LOD) of 3.55 ag/mL. LOD in attomolar range showed an improvement in detection limit compared to previously reported results. This increase in LOD was driven by the use of suspended graphene layer as the channel material in a GFET device. It also demonstrated the ability of the biosensor to detect ADHs below 1 pg/mL, thereby restoring the kidney’s capacity for future applications in an artificial kidney. The immobilization of the ADH-specific aptamer on the graphene layer also restores the $V_{2}$ receptor function in the ADH biosensor. The specificity analysis also revealed that ADH-specific aptamers are highly specific and selective to bind with ADH spiked in PBS and human serum compared to other biomolecules. It can thus be concluded that an ultrasensitive, highly selective ADH sensor with low detection limit for rapid ADH detection has been successfully developed. Laboratory evidence from this work provides promising results which encourage future works related to in vivo testing of ADH within the human body.

## Figures and Tables

**Table 1:** Raman spectroscopy data for graphene on copper.

	$\mathrm{Peak}\;G\;(cm^{-1})$	$\mathrm{Peak}\;2D\;(cm^{-1})$	$\frac{I_{2D}}{I_{G}}$
Point 1	1580.00	2670.00	1.92
Point 2	1580.00	2670.00	2.53
Point 3	1590.69	2674.49	2.22
**Average**	**1582.98**	**2666.78**	**2.21**

**Table 2:** Raman spectroscopy data for graphene on FET.

	Peak D	Peak G	Peak 2D	$\frac{I_{2D}}{I_{G}}$	$\frac{I_{D}}{I_{G}}$
Point 1	1332.28	1581.05	2670.63	2.84	0.19
Point 2	1336.13	1581.05	2674.49	2.22	0.46
Point 3	1338.06	1579.12	2670.63	2.70	0.38
**Average**	**1338.06**	**1581.05**	**2668.70**	**2.58**	**0.34**
